# A Curious Case of Parasitic Fibroid in a Postmenopausal Woman

**DOI:** 10.7759/cureus.25048

**Published:** 2022-05-16

**Authors:** Archana Barik, Vinita Singh

**Affiliations:** 1 Obstetrics and Gynecology, Tata Main Hospital, Jamshedpur, IND

**Keywords:** postmenopausal, extrauterine, wandering, ectopic, parasitic, leiomyoma, fibroid

## Abstract

Parasitic fibroids or leiomyomas are rare extrauterine benign tumors in women of reproductive age. Often, they are named wandering fibroids or ectopic fibroids. They lack any myometrial connection and obtain their nourishment from other abdominopelvic structures to which they are attached. Clinicians often find it difficult to diagnose these fibroids preoperatively due to their atypical presentations and locations. Recent studies have suggested that the development of parasitic fibroids is iatrogenic. Inadvertent seeding of fibroid fragments during the morcellation procedure in a previous laparoscopic myomectomy surgery could be the pathogenesis. However, in rare scenarios, they may develop spontaneously with no history of surgery or a coexistent uterine fibroid. In this report, we present a case of parasitic fibroid in a 75-year-old postmenopausal woman. She had no surgical history, and she had a normal uterus. Radiological investigations had initially suggested the mass to be a subserous fibroid. However, it was diagnosed as parasitic fibroid intraoperatively, confirmed later by histopathological examination.

## Introduction

Uterine fibroid or leiomyoma is one of the common benign tumors of women in the reproductive age group, with an estimated prevalence ranging from 4.5% to 68.6% [1]. They are characterized by the proliferation of smooth muscles and connective tissues of the uterus. Uterine fibroids are conventionally classified as subserous, intramural, or submucous based on their location relative to the different layers of the uterus [2]. Moreover, the International Federation of Gynecology and Obstetrics (FIGO) leiomyoma subclassification system categorizes fibroids into eight subclasses from type 1 to 8 [3]. Type 8 is an additional subtype that recognizes parasitic fibroids as the extrauterine variant. As the name suggests, these fibroids do not have any direct attachment to the uterus, and they receive nourishment from other abdominopelvic structures to which they have adhered [4].

Parasitic fibroids can be primary, secondary, or iatrogenic. Primary variants are presumed to be the detached pedunculated subserous fibroid. They subsequently get adhered to the nearby extrauterine structures and lose any vascular connection with the uterus. Literature regarding primary parasitic fibroids is scarce [5,6]. Instead, several studies have proposed the iatrogenic hypothesis [7-11]. The latter theory postulates that parasitic fibroids develop from the unintentional seeding of tissue fragments generated during the morcellation procedure in a previous laparoscopic myomectomy surgery [12]. Nevertheless, de novo development of fibroids due to metaplastic changes in extrauterine smooth muscle tissues has also been suggested in some rare clinical variants [13].

Uterine fibroids are slow-growing tumors, and they usually shrink after menopause due to the lack of estrogen and progesterone. Therefore, parasitic fibroids are highly unusual in postmenopausal women. This case report discusses the preoperative diagnostic conundrum of an abdominal mass in a postmenopausal woman who presented with abdominal discomfort. It was diagnosed intraoperatively as parasitic fibroid and later confirmed by histopathological examination. She had no history of any abdominal surgery.

## Case presentation

A 75-year-old postmenopausal woman was evaluated at the gynecology clinic for pain and heaviness in the lower abdomen for four months. She was para four, live four, and all her deliveries were vaginal; the last childbirth was 35 years back. She did not have any known comorbidity and had no history of surgery. She had no family history of malignancy or any significant comorbidity. There was no history of any abnormal vaginal discharge or bleeding.

On examination, her vitals were found to be stable. The abdomen was slightly distended, and on palpation, a firm non-tender mass was felt occupying the right iliac fossa and suprapubic area. The mass had a smooth surface, and its movement was restricted from side to side. The liver and the spleen were palpated as normal. There were no perceivable ascites. On per speculum examination, the cervix and the vagina were found to be healthy. The per vaginal examination revealed a mass corresponding to a 20-week size uterus, which felt like a right adnexal mass. The right fornix felt shallow, and the left fornix was normal.

**Figure 1:**
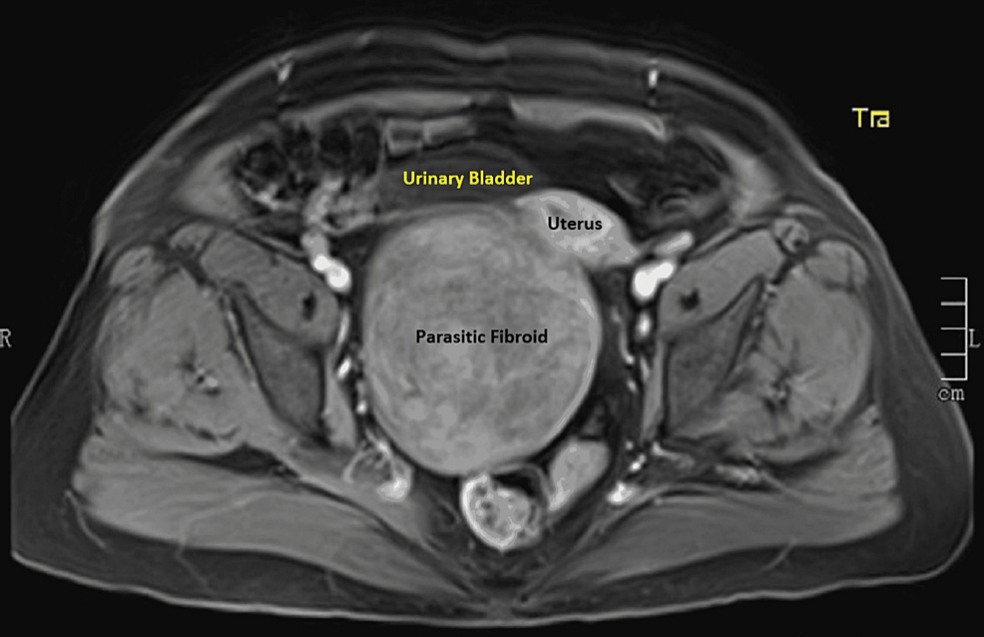
MRI of the abdomen and pelvis (T1-weighted axial image) showing parasitic fibroid, uterus, and urinary bladder. MRI: magnetic resonance imaging

**Figure 2:**
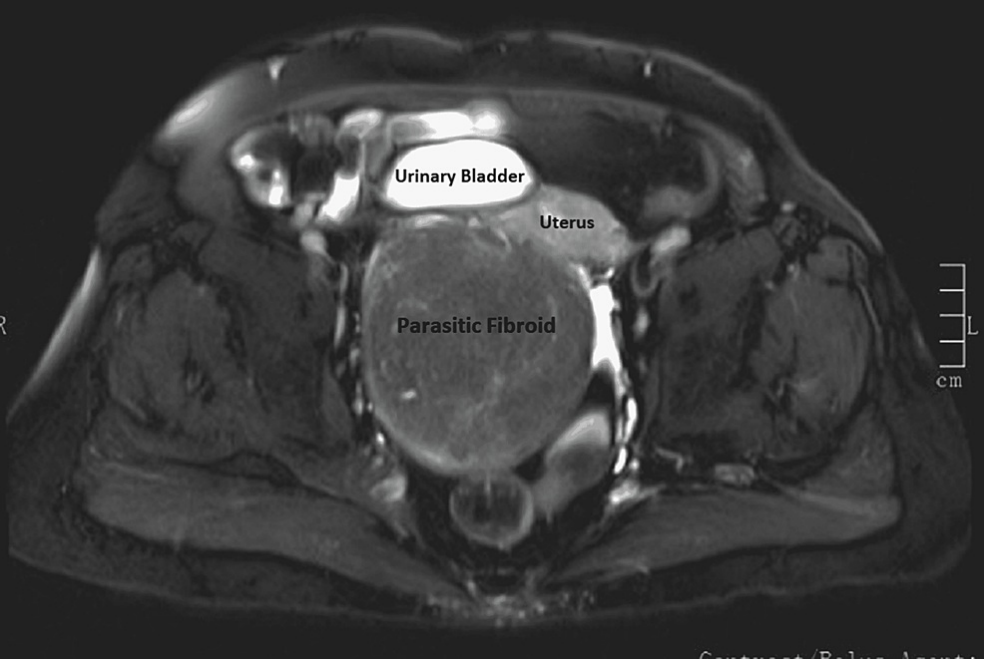
MRI of the abdomen pelvis (T2-weighted axial image) showing parasitic fibroid, uterus, and urinary bladder. MRI: magnetic resonance imaging

**Figure 3:**
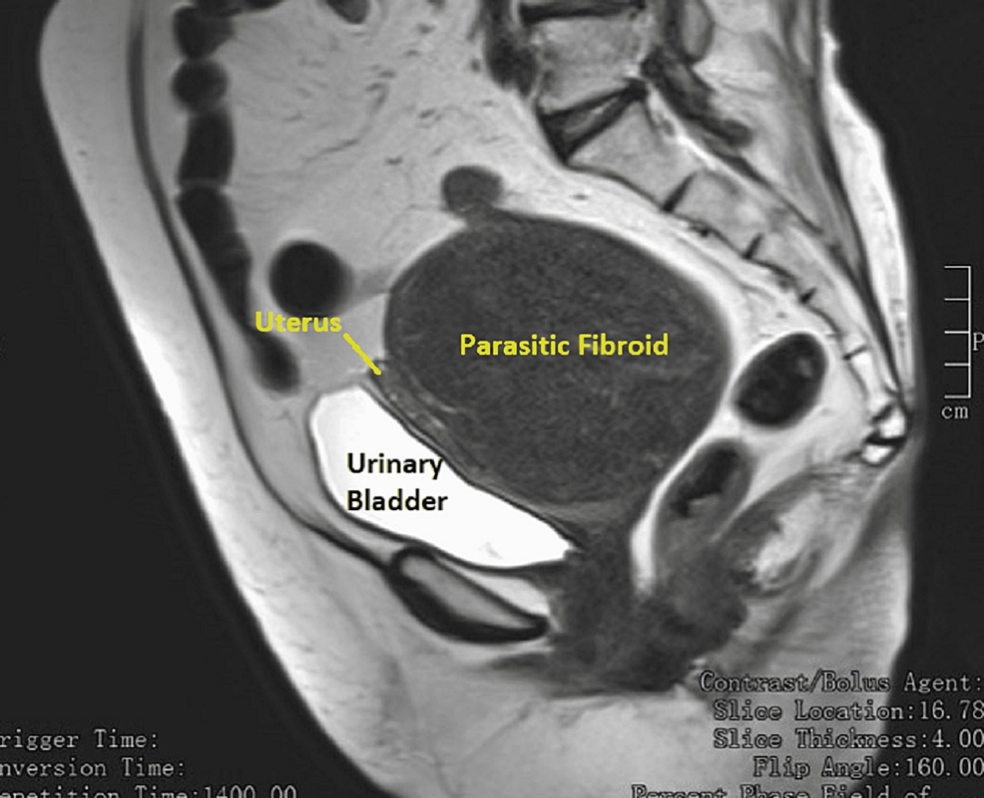
MRI of the abdomen pelvis (T2-weighted sagittal image) showing the relationship between the parasitic fibroid, uterus, and urinary bladder. MRI: magnetic resonance imaging

The hemogram, serum electrolytes, urea, creatinine, liver function tests, and urinalysis were all within normal limits. The serum cancer antigen 125 was 9.4 IU/ml. The chest X-ray and electrocardiogram were normal. Abdominopelvic ultrasonography showed a large heteroechoic solid lesion measuring 11 cm × 7 cm seen involving the posterior myometrium with calcification. The impression was of a large, calcified fibroid in the uterus. The ovaries could not be visualized, and other abdominal organs appeared normal. There were no ascites. Magnetic resonance imaging (MRI) of the abdomen and pelvis revealed a large well-defined encapsulated heterogenous lesion measuring 10.7 cm × 9.5 cm × 9.4 cm noted in the subserous plane of the uterus, displacing it to the left anterolateral aspect (Figures 1-3). The mass was compressing the cervix causing a mass effect and giving the impression of a subserous fibroid.

**Figure 4:**
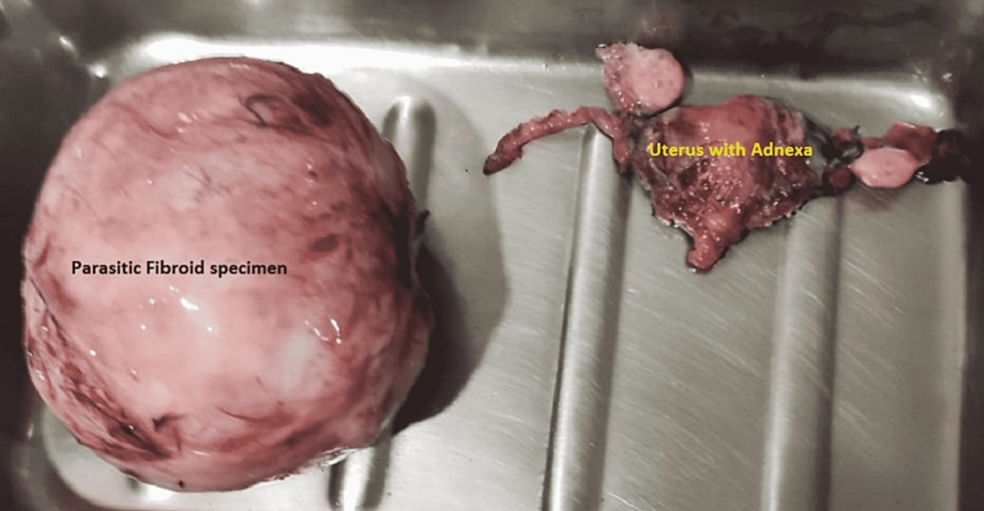
Operated specimen of fibroid along with the uterus. The size of the fibroid was 10 cm × 10 cm × 8 cm.

She underwent an exploratory laparotomy. Intraoperatively, a mass measuring approximately 10 cm × 10 cm × 8 cm with cystic and solid areas was observed. It was filling up the pouch of Douglas and extending up to the right iliac fossa. The mass was completely free from the uterus and was found to have adhered to the right infundibulopelvic ligament. It was also attached to the sigmoid colon and lateral pelvic wall with flimsy adhesions. The uterus was approximately 10 weeks in size, and the fallopian tubes and ovaries appeared normal. All the adhesions were removed, and the mass was excised, followed by total abdominal hysterectomy and bilateral salpingo-oophorectomy (Figure 4).

The patient had an uneventful postoperative course. The histopathology report later confirmed the mass to be a fibroid.

## Discussion

The first reference to parasitic fibroid was by Kelly and Cullens in 1909. They described it as a “myoma that has for some reason become partially or almost completely detached from the uterus and receive their main blood supply from another source” [9]. Since then, most of the literature evidence has emerged from isolated case reports or case series. There are few hypotheses regarding the pathogenesis of this rare clinical condition. Initially, parasitic fibroids were thought to be detached pedunculated subserous uterine fibroids that wandered off and attached to nonuterine tissues. Hence, its often named “wandering or migrating leiomyoma.” In the last few decades, the iatrogenic theory of etiopathogenesis has emerged. The iatrogenic theory proposed that parasitic fibroids originated from the seeding of the leftover tissues of fibroids generated during previous laparoscopic myomectomy. The morcellation procedure performed during myomectomy significantly increases the chances of dissemination of small fragments of fibroid tissue that get implanted onto the surrounding tissues [7-12].

A retrospective analysis of 12 patients with parasitic fibroids by Kho and Nezhat revealed that 10 out of 12 cases had prior abdominal surgeries. They also found that in eight cases morcellation procedure was performed during myomectomy [14]. Lu et al. in their retrospective study of six patients with parasitic fibroids reported that all patients had a history of laparoscopic hysterectomy or myomectomy with the use of a power morcellator [15]. Similarly, Gaspare and colleagues reported an incidence of 0.9% of parasitic fibroids in a retrospective analysis of 423 cases in whom power morcellation was performed during myomectomy [16]. Till now, the literature evidence suggests that parasitic fibroid is always associated with a uterine fibroid. The latter either gets spontaneously detached or inadvertently spread during myomectomy surgery. Rarely, the isolated parasitic fibroid may exist, as seen in a few cases that include the current patient, suggesting that the presence of uterine fibroid or its prior surgery may not always be a prerequisite. However, the age-old hypothesis of detached pedunculated subserous fibroid can be a possibility in the present case.

Theories of de novo development of fibroids have also been described in some rare variants of extrauterine fibroids, such as in leiomyomatosis peritonealis disseminata (LPD) and benign metastasizing leiomyomatosis. LPD is a benign condition characterized by the development of multiple fibroid nodules on peritoneal surfaces. There are multiple theories behind the pathogenesis of LPD. One of them is the estrogen-induced metaplasia and differentiation of subperitoneal mesenchymal stem cells to smooth muscle cells. Similarly, in cases of metastasizing leiomyomatosis, embolization of fibroid tissue from the uterus is implicated as one of the etiopathogeneses [13].

The most likely location of parasitic fibroids is in the pelvis which is the area close to the uterus. Nevertheless, they are seen all over the abdominal cavity. Unusual locations such as the lungs, bladder, urethra, and sigmoid colon have also been reported [13,14,17]. The symptoms associated with parasitic fibroid are usually vague and related to the pressure effect such as pain and heaviness in the abdomen. Patients may present with uterine bleeding, and if the fibroid is large, it may present as a mass abdomen. Often, clinicians face a dilemma in diagnosing parasitic fibroids because of their rarity, aberrant location, and atypical presentation. A detailed medical history and radiological investigations can help clinicians in differentiating parasitic fibroids from other abdominopelvic masses.

Uterine fibroids are tumors of the reproductive age group as their development and proliferation depend on ovarian hormones. As expected, parasitic fibroids are commonly reported in premenopausal women and are extremely unusual in postmenopausal women. In the literature, there were reports of only two cases of parasitic fibroid in postmenopausal women, and in both cases, there were coincident uterine fibroids [6,18]. In the current case, an isolated parasitic fibroid was detected, attached to the infundibulopelvic ligament, and the uterus was normal.

Management of parasitic fibroids is usually surgical either by laparoscopy or open surgery. Medical treatment with gonadotropin-releasing hormone analog was attempted in a few cases with good results but needs further investigation as a treatment modality [19].

Multiple pathological descriptions of extrauterine fibroids or parasitic fibroids have been reported, including LPD, metastasizing leiomyomatosis, ectopic fibroid, etc. The FIGO leiomyoma classification system has added a separate category, type 8, to include all extrauterine fibroids that do not relate to the myometrium, such as cervical fibroids, broad ligament fibroids, and other parasitic lesions [3]. However, considering the literature evidence till now, there is a need to devise a comprehensive nomenclature system to include all the variants of parasitic fibroids.

## Conclusions

Parasitic fibroids are extrauterine fibroids with no myometrial connection. Clinicians often encounter difficulty in diagnosing these fibroids preoperatively due to their rarity, aberrant locations, and unusual presentations. Parasitic fibroids are primarily thought to be a derivative of uterine fibroids. Recent evidence has suggested a higher risk of occurrence after laparoscopic myomectomy using a power morcellator. Rarely, as witnessed in the current case, it may be encountered isolated without any history of myomectomy or coexistent uterine fibroid. This report also demonstrated the persistence of fibroid even after menopause which is uncommon and needs further research.

Clinicians should be highly suspicious of parasitic fibroids due to their tendency to mimic other pelvic tumors. It is rational to keep this rare condition as a differential diagnosis of unusual abdominopelvic mass.
